# Epithelial senescence in idiopathic pulmonary fibrosis is propagated by small extracellular vesicles

**DOI:** 10.1186/s12931-023-02333-5

**Published:** 2023-02-14

**Authors:** Sabha Asghar, Susan Monkley, David J. F. Smith, Richard J. Hewitt, Ken Grime, Lynne A. Murray, Catherine L. Overed-Sayer, Philip L. Molyneaux

**Affiliations:** 1 Bioscience COPD/IPF, Research & Early Development, Respiratory & Immunology, BioPharmaceuticals R&D, AstraZeneca, Cambridge, UK; 2 Translational Sciences & Experimental Medicine, Research & Early Development, Respiratory & Immunology, BioPharmaceuticals R&D, AstraZeneca, Gothenburg, Sweden; 3 Bioscience COPD/IPF, Research & Early Development, Respiratory & Immunology, BioPharmaceuticals R&D, AstraZeneca, Gothenburg, Sweden; 4 National Heart & Lung Institute, Imperial College London, London, UK; 5 Royal Brompton & Harefield Hospitals, Guy’s & St Thomas’ NHS Foundation Trust, London, UK

**Keywords:** Small extracellular vesicles, Exosomes, miRNA, Senescence, Idiopathic pulmonary fibrosis

## Abstract

**Background:**

Idiopathic pulmonary fibrosis (IPF) is a chronic lung disease that affects 3 million people worldwide. Senescence and small extracellular vesicles (sEVs) have been implicated in the pathogenesis of IPF, although how sEVs promote disease remains unclear. Here, we profile sEVs from bronchial epithelial cells and determine small RNA (smRNA) content.

**Methods:**

Conditioned media was collected and sEVs were isolated from normal human bronchial epithelial cells (NHBEs) and IPF-diseased human bronchial epithelial cells (DHBEs).

**Results:**

Increased sEV release from DHBEs compared to NHBEs (n = 4; p < 0.05) was detected by nanoparticle tracking analysis. NHBEs co-cultured with DHBE-derived sEVs for 72 h expressed higher levels of SA-β-Gal and γH2AX protein, p16 and p21 RNA and increased secretion of IL6 and IL8 proteins (all n = 6–8; p < 0.05). sEVs were also co-cultured with healthy air–liquid interface (ALI) cultures and similar results were observed, with increases in p21 and p16 gene expression and IL6 and IL8 (basal and apical) secretion (n = 6; p < 0.05). Transepithelial electrical resistance (TEER) measurements, a reflection of epithelial barrier integrity, were decreased upon the addition of DHBE-derived sEVs (n = 6; p < 0.05). smRNA-sequencing identified nineteen significantly differentially expressed miRNA in DHBE-derived sEVs compared to NHBE-derived sEVs, with candidate miRNAs validated by qPCR (all n = 5; p < 0.05). Four of these miRNAs were upregulated in NHBEs co-cultured with DHBE-derived sEVs and three in healthy ALI cultures co-cultured with DHBE-derived sEVs (n = 3–4; p < 0.05).

**Conclusions:**

This data demonstrates that DHBE-derived sEVs transfer senescence to neighbouring healthy cells, promoting the disease state in IPF.

**Supplementary Information:**

The online version contains supplementary material available at 10.1186/s12931-023-02333-5.

## Introduction

Idiopathic pulmonary fibrosis (IPF) is a chronic, progressive, and ultimately fatal fibrotic lung disease that is characterised by epithelial injury, fibroblast activation, extracellular matrix (ECM) deposition and remodelling [1]. Despite affecting 3 million people worldwide, the cause of IPF remains unknown and current therapies are unable to reverse or even arrest disease progression [1]. There is now growing evidence indicating that IPF involves an aberrant airway epithelial response which contributes to disease progression [2]. Recent papers have noted changes in the morphology of bronchioles and highlighted prominent roles for bronchial epithelial cells in IPF [3–10]. Single nucleotide polymorphisms conferring a higher risk for IPF in several genes—including MUC5B, DSP, FAM13A and AKAP13 – are expressed in bronchial airway epithelial cells [7–13]. Here, we further investigate the role of bronchial epithelial cells in IPF disease progression.

Despite an incomplete understanding of the pathological mechanisms underlying IPF, recent research has highlighted a role for cellular senescence. Senescence is a process by which cells experiencing chronic stress adopt a phenotype characterised by permanent cell cycle arrest, morphologic and gene expression changes and a senescence-associated secretory phenotype (SASP), which triggers a permanent DNA damage response [14, 15]. In IPF and fibrotic disease models, markers of senescence are increased in fibroblasts and  epithelial cells [14, 16]. These cells are thought to promote disease via the SASP, which includes cytokines, proteases and small extracellular vesicles (sEVs, also known as exosomes) [16].

sEVs are 100 nm to 250 nm-sized vesicles, which are a subset of microvesicles released from cells by exocytosis. They are components of the SASP and can transfer their contents to recipient cells and affect neighboring cell phenotype. sEVs have been reported to play roles in multiple physiological processes including apoptosis, angiogenesis, inflammation, coagulation and transfer of cargo such as proteins, lipids and RNA to modulate cellular communication and epigenetic modifications [17]. sEVs contain mRNA transcripts and miRNA as well as small non-coding RNA species, repeat sequences, structural RNAs, tRNA fragments, vault RNA, Y RNA and small interfering RNAs [18]. There is selective incorporation of small RNA species into sEVs, and specific proteins and miRNA are included in sEV cargo depending on cell function and type [19, 20]. Of particular interest is the presence of miRNA within sEVs. Indeed, the majority of circulating miRNA is believed to be sequestered within these vesicles which offer protection from circulating RNases [21, 22]. miRNAs within sEVs can therefore be transported to recipient cells and miRNA content can act by regulating and fine-tuning gene expression to alter target cell phenotype [23, 24].

Differences in specific miRNA expression profiles have been observed between healthy and IPF disease states [25–27]. The miRNA expression profiling of both sputum and serum-derived sEVs from IPF and healthy patients revealed several differentially expressed miRNA [25, 26]. Similarly, distinct sEV miRNA profiles from both bronchoalveolar lavage fluid (BALF) and lung tissue of patients with IPF compared to healthy patients exist [28]. sEVs have also been investigated functionally, with pro-fibrotic and senescent characteristics investigated [27, 29]. IPF-derived sEVs from BALF and primary human lung fibroblasts can mediate WNT5A signalling in IPF [29] and IPF fibroblast-derived sEVs can induce senescence in epithelial cells [27]. In contrast, potential therapeutic uses of sEVs in this context have also been investigated with NHBE-derived sEVs inhibiting transforming growth factor-beta (TGF-*β*) induction of both myofibroblast differentiation and lung epithelial cellular senescence [30].

There remain several unanswered questions regarding the role of senescence in IPF, including the role of the epithelium, the likely site of any initiating injury. We hypothesised that the damaged epithelium in IPF may produce sEVs which could trigger and subsequently propagate a senescent signal. We aimed to characterise and profile sEVs from normal human bronchial epithelial cells (NHBEs) and IPF-diseased human bronchial epithelial cells (DHBEs) and determine the effect of potentially regulatory RNA content on NHBEs and healthy air–liquid interface (ALI) cultures.

## Methods

### Cell culture and sample characterisation

Ethical approval for the study was granted by the Research Ethics Committee (10/HO720/12 and 15/SC/0101). Consent was obtained from all surgical patients for tissue donation for biomedical research purposes. Control participants had no history of respiratory diseases. Characteristics of these cells are detailed in Additional file 1: Table S1. NHBEs and DHBEs were isolated from bronchial brushings from healthy controls and IPF patients undergoing bronchoscopy at the Royal Brompton Hospital (London, UK). For cell isolation, the bronchial brushes were agitated in media to detach the cells and centrifuged at 315 ×*g* for 5 min to pellet the cells. Cells were cultured in Lonza BEBM media + BEGM bullet kit. Media was changed upon reaching 80% confluency and conditioned media removed for sEV collection after 72 h. Cells were used between passages 1 and 3.

### sEV isolation and characterisation

sEVs were isolated by ultracentrifugation (UC) or ExoQuick (Systems BioScience). For UC, media collected from NHBEs and DHBEs underwent differential centrifugation at 350 ×*g* for 10 min to remove large debris and floating cells and 2000 ×*g* for 10 min, to remove large cells and apoptotic bodies. Media was transferred to UC tubes and spun at 100,000 ×*g*/45,000 RPM at 4 °C for 1 h (Beckman Coulter Optima LE-80 K ultracentrifuge; Ti45.1 Beckman Coulter fixed angle rotor). The supernatant was removed and spin repeated in PBS, before resuspension of the pellet in 100 μl PBS. This method was used for initial NTA experiments before comparisons with ExoQuick. ExoQuick usage yielded comparable amounts with shorter protocol length. Subsequent experiments therefore utilised ExoQuick. The ExoQuick methodology was carried out according to the manufacturer’s protocol. Briefly, 1 ml of ExoQuick- TC/5 ml media was added and refrigerated overnight before centrifugation at 1500 ×*g* for 30 min and aspiration of 95% of supernatant. A further spin at 1500 ×*g* for 5 min was undertaken, before aspiration of remaining liquid. Pellets were resuspended in sterile phosphate-buffered saline (PBS), laemmli sample buffer (Bio-Rad) or exosome resuspension buffer (Life Technologies), as specified in the text.

Nanoparticle tracking analysis (NTA) was carried out using the NS300 (Malvern Instrumentation) with samples injected via a flow-through syringe into the viewing chamber. The NTA machine uses a light scattering methodology to extract particle size and the number of particles in a sample. The NTA software collects data on multiple particles simultaneously and calculates the hydrodynamic diameter of each particle using the Stokes–Einstein equation.

Transmission electron microscopy (TEM) images were captured using the JEOL JEM-1400 on the AMT XR16 ActiveVu camera system. sEV preparations were resuspended in 4% PFA and 5 μl of the suspension was pipetted onto clean parafilm with electron-microscope grids placed on top (coated side facing the suspension). PBS (100 μl) was subsequently added drop-wise onto the parafilm and grids for washing. To the grids, 50 μl of 1% glutaraldehyde was added for 5 min followed by multiple washes in distilled water. Grids were then transferred to a 50 μl drop of uranyl-oxalate solution, pH 7, for 5 min and a 50 μl drop of methyl cellulose-UA for 10 min on ice. The grids were removed with stainless steel loops and excess fluid removed onto filter paper before air drying and imaging.

sEV markers were blotted using a standard western blot methodology with a specialised Exosomal Marker Antibody Sample Kit (Cell Signalling Technology, #74,220), using the antibodies detailed in Additional file 1: Table S2. Briefly, samples were boiled at 90 °C for 5–10 min before being loaded at a concentration of 20 μg/ml. 5 μl of Amersham ECL Full-Range Rainbow Molecular Weight Marker (Cytiva #RPN800E) were loaded as a ladder. Samples were run on Bolt 4–12% Bis–Tris Plus 12-well gels (Invitrogen) NuPAGE MOPS SDS running buffer (Life Technologies). Gels were transferred to nitrocellulose membranes (Bio-rad) using the Trans-Blot Turbo transfer pack 0.2 μm Nitrocellulose (Bio-rad). Membranes were blocked with PBS Tween20/5% milk before incubating with primary antibodies overnight at 4 °C. The membrane was then incubated with a secondary antibody for 1 h before covering in 1.5 ml Clarity max (high sensitivity) ECL substrate and 1.5 ml ECL enhancer and reading on the Licor c-Digit imager (Licor) with c-digit ImageStudio software. Membranes were washed between each step with PBS/Tween20. Cell lysates and lysis buffer were used as controls in these experiments.

ExoELISAs (EXO series, SystemsBioscience) were carried out according to the manufacturer’s protocol. ExoELISA quantification is proportional to the amount of specific antigen present in each well. Exosome binding buffer was added to the sEV pellet before incubating at 37 °C for 20 min to liberate exosome proteins. 50 µl of freshly prepared protein standards and sEV samples were pipetted into the appropriate well of a micro-titer plate and incubated at 37 ºC for 1 h. 50 μl of primary antibody diluted in block buffer was added to each well and incubated for 1 h. 50 μl of diluted secondary antibody was then added and incubated for 1 h with shaking before the addition of super-sensitive TMB ELISA substrate. After incubation at room temperature for 15 min with shaking, 50 µl of stop solution was added and the plate read immediately on the Cytation5 (BioTek) at 450 nm. Plates were washed between each step with wash buffer on a shaker.

### Fluorescent uptake

Uptake was visualised using the ExoGlow-RNA labelling kit (Systems Bioscience) as per the manufacturer’s protocol. Briefly, labelling reaction buffer was added to 50 μg sEVs and incubated overnight before removal of the free probe by centrifugation and co-culture with epithelial cells. Cells were incubated for between 0 and 72 h at 37 °C and 5% CO_2_, before visualisation by confocal microscopy. Quantification and imaging were also undertaken on the Cytation5 (BioTek), with four high-power fields evaluated firstly for cell number (phase contrast) and then fluorescent intensity.

### Co-culture experiments

sEVs were quantified with the Pierce BCA Protein Assay Kit (Thermo Fisher) and used at a concentration of 10 μg/ml for 72 h (as used in Kadota et al. [27]). In brief, standards were prepared between 0–2000 μg/ml and 10 µl transferred to 96-well nunc clear plates in duplicate. Samples were also added in duplicates to the plate. Working reagent (50:1 reagent A: reagent B) was prepared and 200 µl added per well. A clear plate sealer was added and the plate incubated for 30 min at 37 °C, before reading on the Cytation5 (BioTek) at 562 nm.

For submerged cultures, NHBEs were plated in both 6 and 96-well plates at a concentration of 5 × 10^4^ cells/well and 5 × 10^3^ cells/well respectively. NHBE and DHBE-derived sEVs were co-cultured with these cells for 72 h before cells and media harvested.

For ALI culture, transwells (Sigma) were coated overnight at 37 °C and 5% CO_2_ with 200 µl 1 × collagen solution (Stemcell) and washed with 1 × PBS and left to air dry. NHBEs were initiated into EpiX basal medium with supplements (Propagenix) and media changed every other day until cells reached 80% confluency. 30 mM HEPES (Lonza) and 1 × trypLE were used for detachment, before neutralisation with HBSS (with calcium and magnesium) + 5% FBS + Trypsin neutralising solution (TNS) (all Thermo Fisher). Cells were resuspended in PneumaCult™-ALI medium (Stemcell Technologies) at 5 × 10^5^/ml and 0.5 ml added to the transwell. Once cells were confluent, the ALIs were air-lifted by aspirating the apical media and replacing the basolateral media with PneumaCult™-ALI Medium. Once differentiated, 10 μg/ml of sEVs were added apically and left for 72 h before cells and media were harvested. 300 μl of PBS was then added apically to the culture and incubated for 30 min at room temperature before also being harvested for ELISAs.

Cells not incubated with sEVs and cells incubated with sEVs that had been previously incubated with Triton X were included in these experiments as controls. Etoposide was also used in these experiments as a positive control for induction of senescence.

### Transepithelial electrical resistance (TEER)

TEER measurements were taken on ALI cultures before and after stimulation, using an EVOM^3^ epithelial voltometer (World Precision Instruments) to calculate the percentage change in epithelial barrier integrity. ALI cultures were prepared by collecting the basal media and adding 250 μl PBS to the apical compartment and 950 μl PBS to the basal compartment. A blank transwell control with no cells was used for background measurements. TEER measurements were performed by vertically placing the long probe through the insert break into the transwell and lowering until it reaches the bottom, while the short probe rests on the ALI insert.

### γH2AX staining and imaging

Plates were fixed by adding 100 μl/well of 4% formaldehyde (Thermo Fisher), permeabilised using permeabilisation buffer (0.25% Triton/PBS) and blocked using PBS/3%BSA. γH2AX primary antibody (Phospho-Histone H2A.X Rabbit mAb) (Thermo Fisher) was added at a dilution of 1:400 at 4 °C overnight. Incubation with Alexa Fluor 488 goat anti-rabbit IgG (H + L) secondary antibody and DRAQ5 nuclear stain (5 nM) (both Thermo Fisher) took place for 1 h before imaging. 15 mg/ml Rabbit IgG isotype and secondary only wells were also included as controls. Cells were washed in PBS/Tween20 between each of these steps. Imaging for γH2AX positive cells was undertaken on the Cytation5 (BioTek).

### SA-β-Gal staining and imaging

SA-β-Gal staining was performed using the Cellular Senescence Detection Kit—SPiDER-ßGal (Dojindo) as per the manufacturer’s instructions. Briefly, Bafilomycin A1 working solution was added to the plate and incubated at 37 °C and 5% CO_2_ for 1 h. The solution was discarded and replaced with SPiDER-βGal working solution and incubated at 37 °C and 5% CO_2_ for 30 min. Cells were washed twice before staining with Hoechst 33,342 (Invitrogen) and HCS CellMask™ Red Stain (Life Technologies). Plates were incubated at 37 °C and 5% CO_2_ for 1 h and observed under a fluorescent microscope.

### IL6 and IL8 ELISAs

IL6 and IL8 levels were quantified using R&D Systems human duoset ELISAs with a modified europium protocol. Briefly, black nunc plates were coated with capture antibody in coating buffer and incubated overnight at 4 °C. Plates were blocked with PBS/3% BSA before samples and standards added and incubated for 2 h. Detection antibody was then added for 2 h before Streptavidin-Europium diluted 1:1000 in DELFIA® assay buffer (both Perkin Elmer) was added for 30 min. Finally, DELFIA^®^ enhancement solution (Perkin Elmer) was added for 10 min before reading on an Envision 2103 Multilabel Reader (Perkin Elmer). Plates were washed in PBS/Tween20 between each of these steps.

### RNA and cDNA isolation

Total RNA was isolated from cells using the Qiagen RNeasy Plus Kit (Qiagen) using a Qiacube according to the protocol and quantified using the Nanodrop ND-1000 spectrophotometer; Software ND-1000 v3.7.0 (Thermo Fisher). For mRNA expression, cDNA was generated using the High Capacity RNA-to-cDNA kit (Thermo Fisher). Customised Taqman array cards were generated by Thermo Fisher (7427384/B5645). Array cards were brought to room temperature and 100 ng of cDNA loaded for each sample together with TaqMan Universal Master Mix II, no UNG (Thermo Fisher). The plate was centrifuged twice at 314 ×*g* for 1 min before being sealed and run using a QuantStudio 7 Flex and QuantStudio™ Real-Time PCR Software (Thermo Fisher). All mRNA quantification data from cultured cells were normalized to GAPDH housekeeping gene. All miRNA quantification data from cultured cells were normalised to smRNA RNU6B which was confirmed to be stably expressed across generated samples.

For sEV preparations, RNA was isolated using the total exosome RNA and protein isolation kit (Life Technologies) as per the manufacturer’s protocol. RNA was quantified using the Agilent RNA 6000 PICO kit on the Agilent Bioanalyser (Agilent Technologies) as per the manufacturer’s protocol. For miRNA expression, cDNA was prepared using TaqMan Advanced miRNA Assays and TaqMan Advanced miRNA cDNA synthesis Kit (Thermo Fisher). Briefly, a poly(A) tailing reaction, adaptor ligation reaction, reverse transcription and miR-Amp reaction were run. RNA input was normalised for cDNA generation after measurement using the RNA Pico Assay Reagent Kit (Perkin Elmer) and assessment on the Agilent 2100 bioanalyzer (Agilent Technologies) on the pico chips. This template was diluted 1:10 for qPCR reactions. The data were collected and analysed using a QuantStudio 7 Flex and QuantStudio™ Real-Time PCR Software (Thermo Fisher). Primers used for these experiments are shown in Additional file 1: Table S3. There is yet no consensus on the use of normalisation genes for exosomal miRNA, although multiple papers have used RNU6B as an internal reference [31–33]. All sEV miRNA quantification data were therefore normalized to RNU6B, which was stably expressed in these samples. Samples were further normalised to the relevant control group. We have shown absolute quantification as QT values in supplementary data [34] (Additional file 1: Fig. S5).

### smRNA-sequencing and analysis

Quality control of RNA isolated from cells and their derived sEVs was carried out by Source BioScience (Cambridge, UK). Libraries were prepared using the NEBNext^®^ Ultra™ II Directional RNA Library Prep Kit according to the manufacturer’s protocol. During this process, the libraires were indexed using NEBNext^®^ Multiplex Oligos for Illumina^®^. The prepared libraries were quantified via a fluorometric method involving a Promega QuantiFluor dsDNA assay; and qualified using electrophoretic separation on the Agilent TapeStation 4200. All samples were pooled and sequenced on the NovaSeq. Data was returned as raw fastQ data.

Data received was run through a pre-defined bioinformatics pipeline. A smRNA-seq pipeline was implemented which includes quality controls (QC), adapter sequence trimming of raw reads by atropos, STAR to align against the genome (hg38) and other smRNA detection by SeqCluster. miRNAs were detected using miraligner tool with miRBase (21 release, http://www.mirbase.org/) as the reference miRNA database, the quantification of known smRNAs was carried out by SeqBuster. Count matrices were generated for miRNA and isomiRs by combining data for each sample with miRNAs as rows and samples as columns. FastQC was used for QC and multiqc for reporting. The miRNA count matrix was used for differential expression analysis in R. DESeq2 Bioconductor package was implemented in the R/RStudio. Results were later filtered for adjusted p-values (p.adj) threshold (or false discovery rate, FDR) of < 0.05.

Qiagen Ingenuity Pathway Analysis software, Pubmed and DIANA-mirPath software were used to investigate differentially expressed miRNA datasets and mRNA targets. miRNA-mRNA relationships and gene predictions were prioritised based on specific senescence pathways links before visualisation. IPA’s microRNA Target Filter uses content from Tarbase, miRecords, TargetScan and published literature while DIANA-miRpath KEGG and GO analysis takes place using Tarbase.

### Statistics

All datasets are presented as unique donors and experiments. Non-parametric statistical tests were used throughout the paper. Mann–Whitney tests were used for the comparison of two unpaired groups and Wilcoxon test used to compare two paired groups. For multiple comparisons, the Friedman test or the Kruskal–Wallis test was used. Significance was defined as P < 0.05 and statistical software used was Prism version 6 (GraphPad Software, San Diego, CA).

## Results

### DHBEs release a higher number of sEVs, compared to NHBEs

**Fig. 1:**
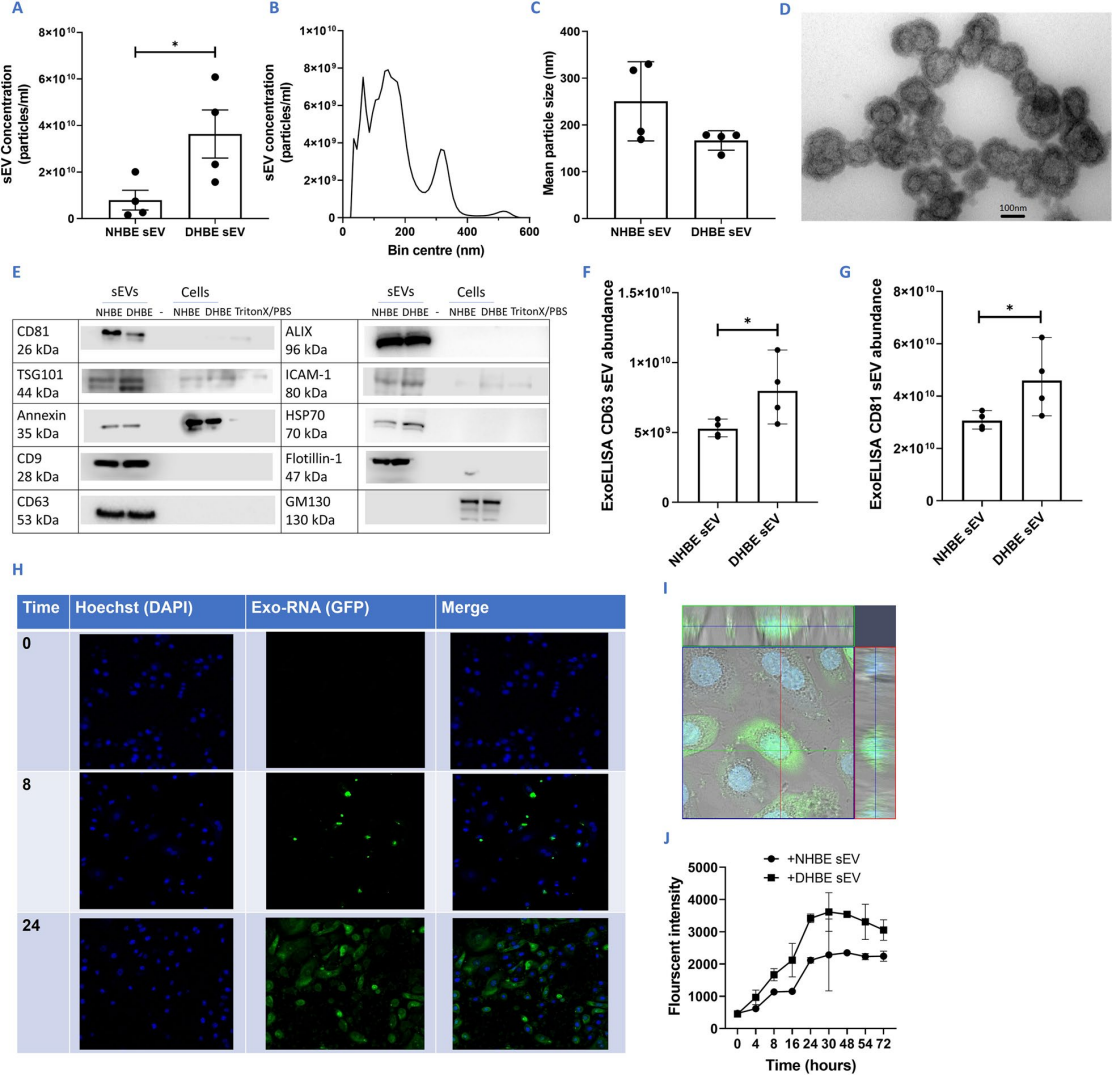
Characterisation and quantification of sEVs from diseased and healthy donors. **A** Increased sEV release from DHBEs. **B** Example of DHBE-derived particle distribution curve from NTA. **C** Mean particle size of sEVs released from NHBEs and DHBEs. **D** Example TEM image of sEVs after isolation. Magnification× 50,000. **E** Western blot of common proteins expressed by sEVs (positive control) and proteins not expressed by sEVs (negative control-GM130) confirming minimal contamination within preparations. Blots have been cropped to relevant lanes and protein size. **F**, **G** ExoELISA quantification showing higher abundance of CD63 and CD81 in DHBE-derived sEV preparations/wells. **H** Example imaging for uptake of DHBE-derived sEVs by NHBEs over 24 h using exo-RNA glow (green) and hoechst (blue) probe. **I** Z-stack image showing uptake of sEVs into cells. **J** Quantification of exo-RNA uptake by responder cells shows increased uptake over time and maximal uptake occurs between 24 and 30 h. N = 4–6. Each dot represents a different donor; A–D isolated by ultracentrifugation, all other experiments by ExoQuick as detailed in methodology. Note, due to the low yields of sEVs each assay was run with an individual sEV preparation. Significant differences between groups shown *p < 0.05, Mann–Whitney test

Increased sEV release from DHBEs compared to NHBEs was detected by nanoparticle tracking analysis (NTA), with an average of 3.64 × 10^10^ particles/ml and 0.794 × 10^10^ particles/ml released respectively (n = 4; p < 0.05) (Fig. 1A). sEV preparations from all sources had a mean particle distribution of between 167 and 250 nm (n = 6) (Fig. 1B, C). The presence of sEVs between 100 and 250 nm was observed by TEM imaging of these preparations. These displayed a double membrane (or depression) and a cup-shaped morphology as expected (Fig. 1D).

sEV preparations were then characterised by western blot, measuring proteins known to be either enriched or present in sEVs, including HSP70, CD9, CD81, CD63, ALIX, Annexin, ICAM-1, Flotillin-1 and TSG101 [17] (Fig. 1E). Parental cells were included as controls and all samples were blotted for GM130 a as a negative control (ordinarily enriched in cells) to ensure minimal contamination from cellular contents during the sEV isolation process. Two common sEV markers—CD81 and CD63—were also quantified using EXOUltra to determine patterns in abundance between healthy and diseased sEV release (Fig. 1F, G). Both markers had increased abundance in DHBE-derived sEV preparations compared to NHBE-derived sEV preparations [average of 7.97 × 10^9^ positive particles/ml and 5.26 × 10^9^ positive particles/ml for CD63 respectively and average of 4.59 × 10^10^ and 3.06 × 10^10^ positive particles/ml for CD81 respectively (n = 4; p < 0.05) (Fig. 1F, G)]. Combined with NTA data, this confirms an increase in the release of particles from DHBEs.

### DHBE-derived sEVs are taken up by NHBEs, with maximal uptake at 24 h

RNA within sEVs were fluorescently tagged and incubated with NHBEs. A time course of images indicated successful sEV uptake, with maximal uptake reached by 24 h (Fig. 1H, J). Z-stack images confirmed the uptake of both NHBE and DHBE-derived sEVs into NHBEs (Fig. 1I).

### DHBE-derived EVs induce a higher degree of senescence in NHBEs, compared to NHBE-derived EVs

**Fig. 2:**
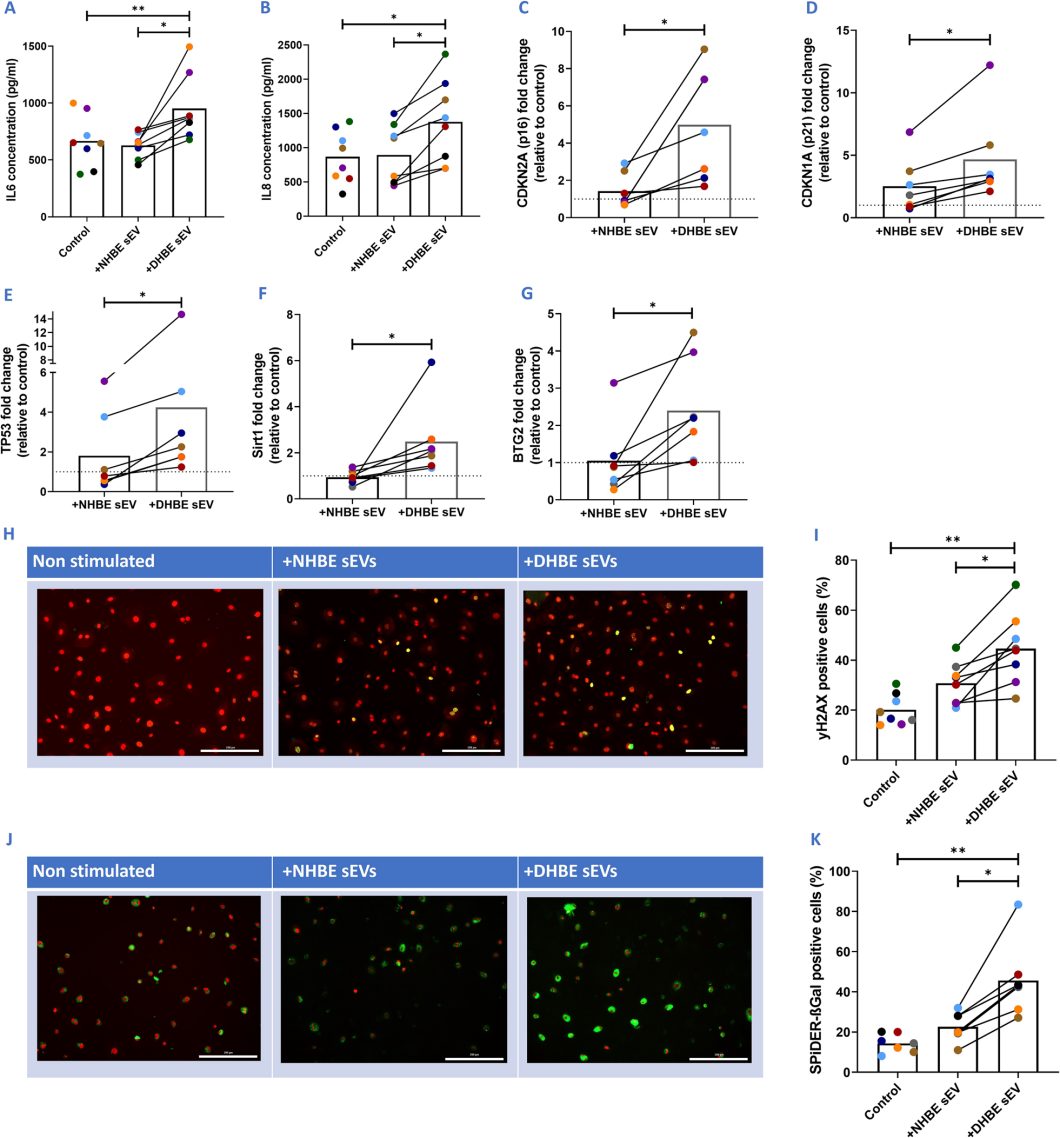
sEVs act as part of the SASP and transfer senescence to co-cultured NHBEs in submerged assays. **A** Increases in IL6 with addition of DHBE-derived sEVs and **B** A similar pattern seen with IL8. **C**–**H** Increased senescence related gene expression with co-culture of DHBE-derived sEVs; **C** p16, **D** p21, **E** TP53, **F** Sirt1, and **G** BTG2 expression increases with addition of DHBE-derived sEVs. Gene normalisation to GAPDH for δδCT and graphs show fold change relative to untreated control group. **H** Representative images of γH2AX (green) and DRAQ5 (red) staining upon addition of NHBE and DHBE-derived sEVs. **I** Quantification of γH2AX showing increased DNA damage with DHBE-derived sEV addition. **J** Representative images of SA-β-Gal (green) and DRAQ5 (red) staining upon addition of NHBE and DHBE-derived sEVs. **K** Quantification of SA-β-Gal showing increased expression with DHBE-derived sEV addition. N = 6–8. Each colour represents a different donor and experiment. Significant differences between groups shown **p < 0.01; *p < 0.05, Wilcoxon test and Friedmans test

To determine whether these sEVs have a functional effect, NHBE and DHBE-derived sEVs were co-cultured with NHBEs for 72 h. Secretion of IL6 and IL8 SASP proteins from NHBEs were increased upon the addition of DHBE-derived sEVs, in comparison to NHBE-derived sEVs (952.0 pg/ml and 626.9 pg/ml respectively for IL6; 1377.4 pg/ml and 893.9 pg/ml respectively for IL8) (N = 8; p < 0.05) (Fig. 2A, B). Gene expression of G1/S cell–cycle checkpoint regulators p16 and p21 were increased with the addition of DHBE-derived sEVs, in comparison to the untreated control (fivefold and 4.6 fold respectively (Fig. 2C, D) (N = 7; p < 0.05). There were also significant increases in other senescence-associated genes such as BTG2, TP53 and SIRT1 (N = 6; p < 0.05) (Fig. 2E–G). γH2AX is a marker of DNA damage and the percentage of γH2AX positive cells were increased with the addition of DHBE-derived sEVs, in comparison to NHBE-derived sEVs (44.6% and 30.7% respectively) and the unstimulated control (20.1%) (N = 8; p < 0.05) (Fig. 2H, I). SA-β-Gal is a specific senescence marker and SA-β-Gal positive cells were increased with the addition of DHBE-derived sEVs in comparison to NHBE-derived sEVs (45.6% and 22.6% respectively) and the unstimulated control (14.3%) (N = 7; p < 0.05) (Fig. 2J, K).

To determine whether these vesicles need to be intact to induce a response, DHBE-derived sEVs were incubated with Triton X, a detergent that breaks down the sEV membrane to release the contents. Triton X-incubated DHBE-derived sEVs failed to induce a senescent signal (Additional file 1: Fig. S2) suggesting that intact sEVs are crucial for this mechanism (N = 3; p < 0.05).

### DHBE-derived sEVs induce a higher level of senescence in healthy ALI cultures, compared to NHBE-derived sEVs

We next assessed the effect of sEVs in an ALI culture model. Like bronchial epithelium, these cells are pseudostratified in morphology and made up of a heterogeneous cell population (including ciliated, basal and mucus secreting cells) [35]. In these cultures, the basal surface of the cells is in contact with liquid culture medium and the apical surface is exposed to air.

**Fig. 3:**
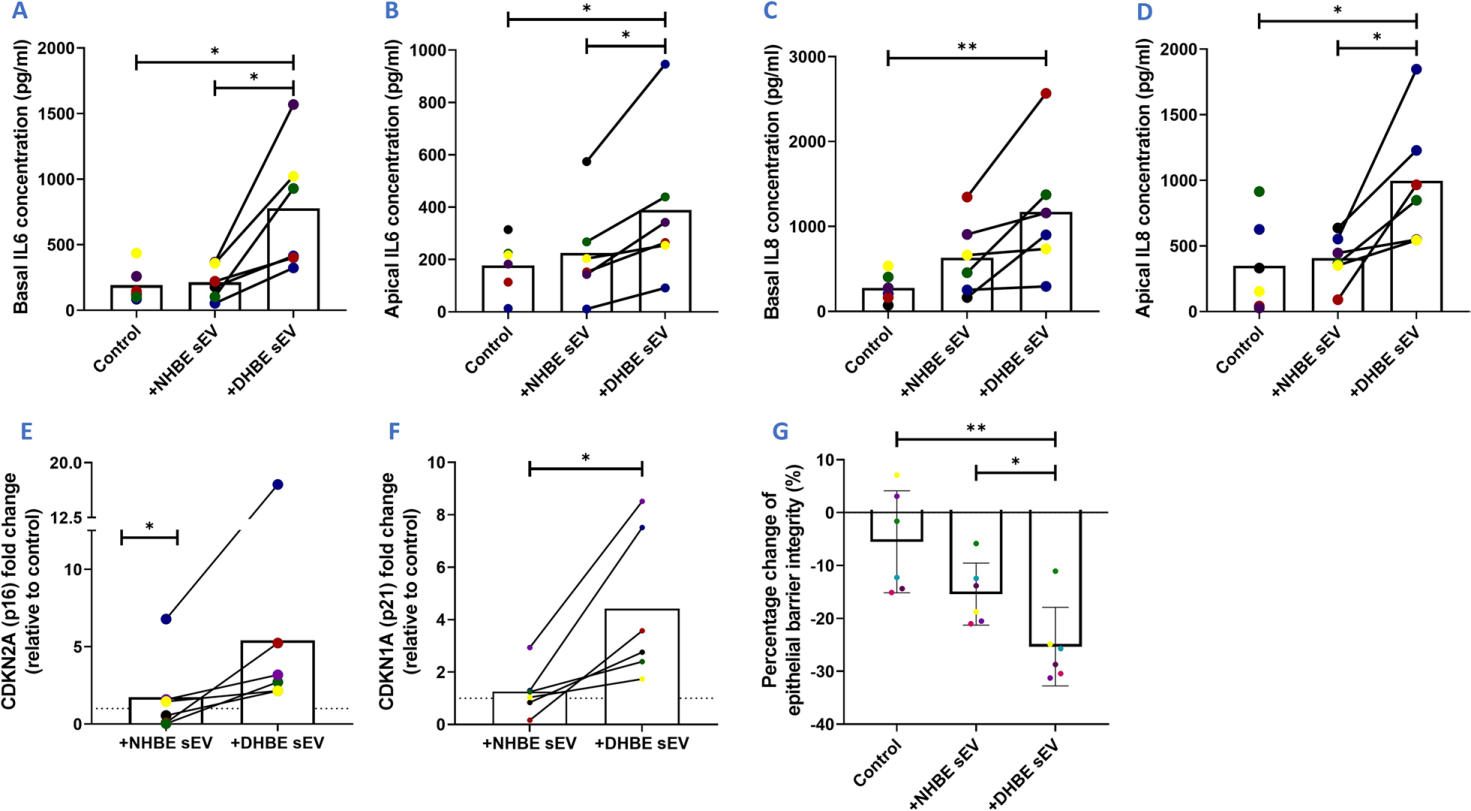
sEVs act as part of the SASP and transfer senescence to healthy ALI cultures. **A**, **B** IL6 levels in basal media and apical PBS wash increased with DHBE-derived sEV addition. **C**, **D** IL8 levels in basal media and apical PBS wash increased with DHBE-derived sEV addition. **E**, **F** Increases in p21 and p16 gene expression upon addition of DHBE-derived sEVs. Gene normalisation to GAPDH for δδCT and graphs show fold change relative to untreated control group. **G** Percentage change in TEER with the addition of DHBE-derived sEVs. N = 6. Each colour represents a different donor and experiment. Significant differences between groups shown **p < 0.01; *p < 0.05, Wilcoxon test and Friedmans test

Secretion of IL6 proteins were increased in both the basal media and apical PBS washes from healthy ALI cultures upon the addition of DHBE-derived sEVs, in comparison to NHBE-derived sEVs (basally 776.9 pg/ml and 214.2 pg/ml respectively; apically 389.6 pg/ml and 225.3 pg/ml respectively) (N = 6; p < 0.05) (Fig. 3A, B). Secretion of IL8 proteins were also increased in both the basal media and apical PBS washes from healthy ALI cultures upon the addition of DHBE-derived sEVs, in comparison to untreated cells (basally 1172.1 pg/ml and 277.2 pg/ml respectively; apically 997.1 pg/ml and 349.2 pg/ml respectively [both n = 6; p < 0.05) (Fig. 3C, D)]. NHBE-derived sEVs did not induce a significant increase in basal or apical IL8. p16 gene expression levels increased 5.4-fold with addition of DHBE-derived sEVs, compared to the untreated control (N = 6; p < 0.05) (Fig. 3E). p21 expression levels increased 4.4-fold with the addition of DHBE-derived sEVs, compared to the untreated control (N = 6; p < 0.05) (Fig. 3F). TEER measurements, an indication of integrity of the ALI epithelial barrier, decreased with the addition of DHBE-derived sEVs in comparison to NHBE-derived sEVs (25.7% and 15.4% respectively) and the unstimulated control (5.52%) (N = 6; p < 0.01) (Fig. 3G, Additional file 1: Fig. S3). This data shows a clear effect of sEVs from DHBEs on the senescent phenotype in ALI culture.

### sEV miRNA profiles indicates active and selective packaging between cells and sEVs

**Fig. 4:**
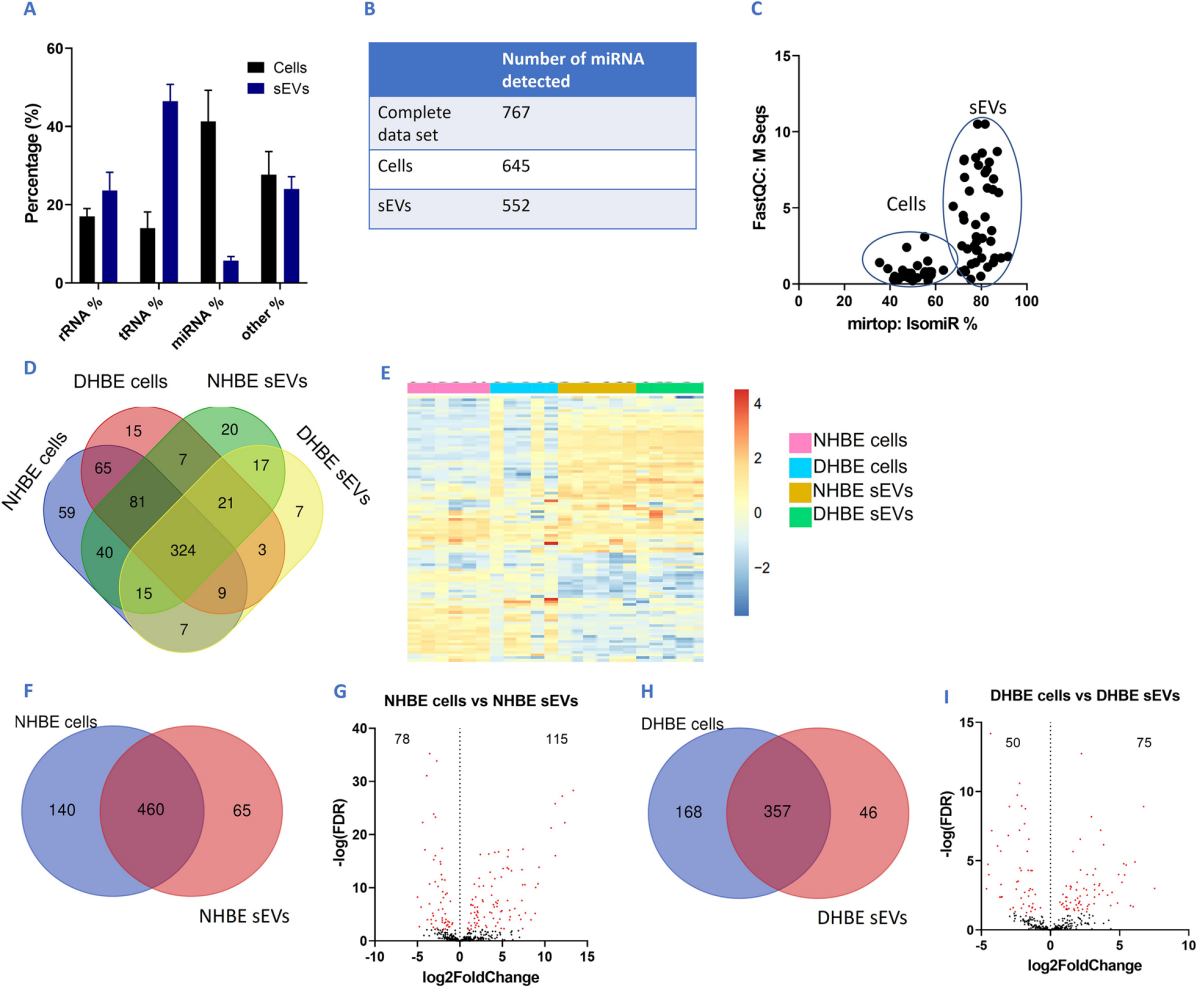
smRNA-seq shows very different composition of smRNAs between cells and sEVs. **A** Percentage of different subsets of smRNA in cells and sEVs, showing widely differing composition (other = other types of smRNA) **B** Volcano plot (log p vs log fold change) showing 747 miRNA variables were found in this complete dataset. **C** IsomiR% (variants of miRNA) and M Seq (sequence count) showing differences in profiles between cells and sEVs and higher variability in sEV samples. **D** Overlap of miRNAs between sample sets. **E** Heat map showing expression level differences between sEVs and cells, including NHBE and DHBEs. **F** Overlap of miRNAs detected in NHBE cells and NHBE sEVs. **G** Volcano plot showing differential miRNA expression between NHBEs and their derived sEVs. **H** Overlap of miRNAs detected in DHBE cells and DHBE sEVs. **I** Volcano plot showing differential miRNA expression between DHBE cells and their derived sEVs. N = 5–6. Red p.adj < 0.05

To determine whether miRNAs within the sEVs were contributing towards the senescent phenotype observed, we profiled the miRNA cargo of these sEVs by performing smRNA-sequencing of parental cells and their derived sEVs. Interestingly, the percentage of smRNA that was characterised as miRNA within sEVs (5.7%) were low in proportion to rRNA (23.6%) and tRNA (46.5%) (Fig. 4A). Overall, 747 miRNAs were detected in this complete dataset (Fig. 4B). Profiles show separation in sEV and cellular profiles and Venn diagrams show less than 50% of miRNA detected in the data set were detected in every group, however very few miRNAs were specific to a particular source (Fig. 4C, D). Heat maps also show the differential expression of miRNA in these sources, with a striking difference between cells and sEVs (Fig. 4E).

Venn diagrams show that out of the 665 miRNA present in either NHBEs and NHBE-derived sEVs, the majority (460) of these were common to both sources (Fig. 4F). However, NHBE-derived sEVs had 193 significantly differentially expressed miRNAs, in comparison to their parental cells; 78 were downregulated and 115 were upregulated (n = 6; p.adj < 0.05) (Fig. 4G). Similarly, venn diagrams show that out of the 571 miRNA present in either DHBEs and DHBE-derived sEVs combined, the majority (357) of these were common to both sources (Fig. 4H). However, DHBE-derived sEVs had 125 significantly differentially expressed miRNAs, in comparison to their parental cells—50 were downregulated and 75 were upregulated (n = 5; p.adj < 0.05) (Fig. 4I). These data indicate that packaging of miRNA into sEVs during sEV formation is an active rather than a passive mechanism, with significantly different miRNA profiles found between cells and their derived sEVs.

### sEV miRNA profiles confirm that differential packaging is dependent on IPF disease state

**Fig. 5:**
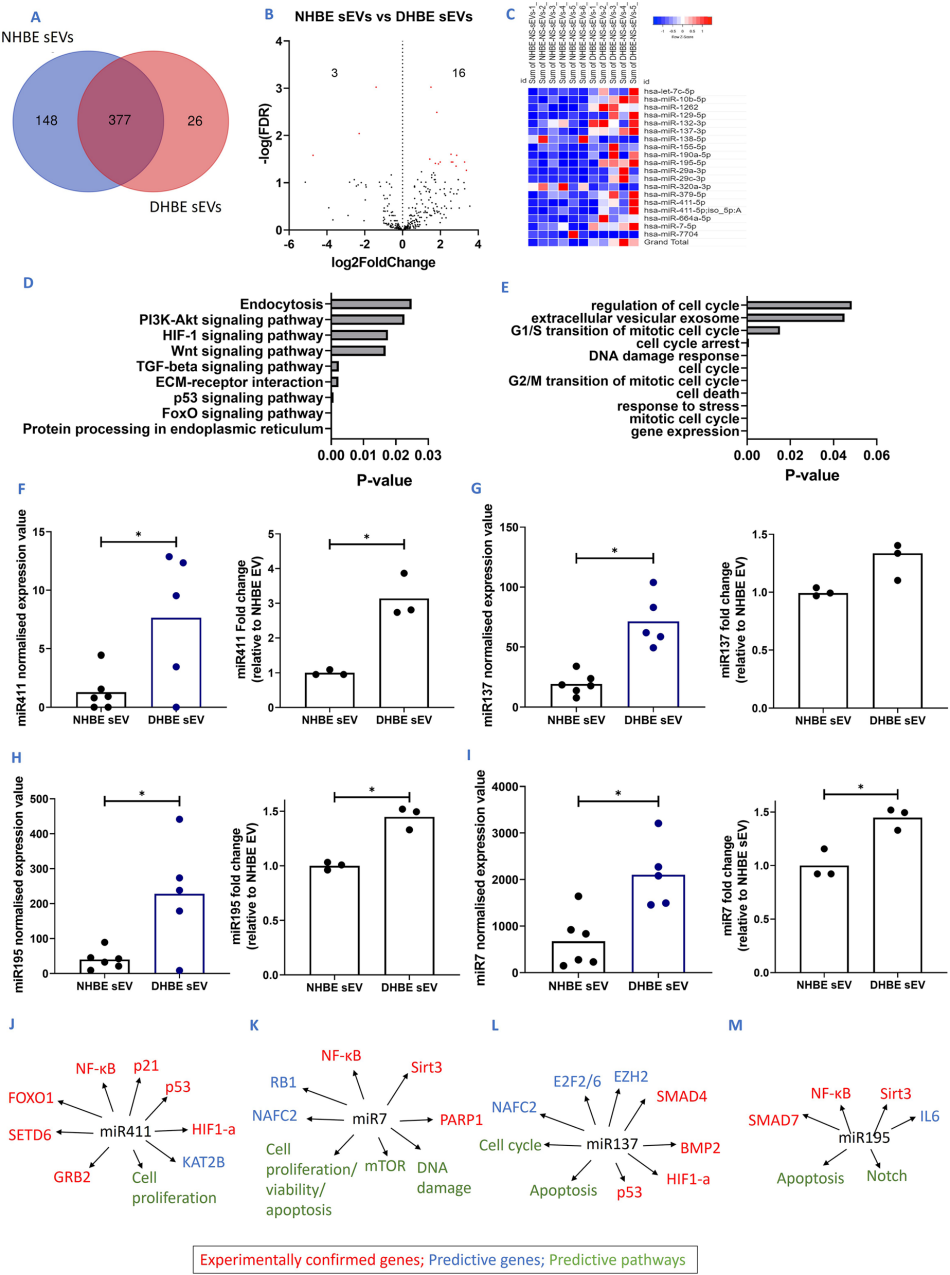
smRNA-seq shows differential miRNA composition of smRNAs between healthy and diseased sEVs. **A** Overlap of miRNAs detected in NHBE sEVs and DHBE sEVs. **B** Volcano plot showing differential miRNA expression between NHBE and DHBE-derived sEVs. **C** Heat map detailing expression differences of miRNA between NHBE and DHBE-derived sEVs. **D** KEGG pathway analysis of differential sEVs. **E** GO pathway analysis of differential sEVs.**F**–**I** smRNA-seq expression values and confirmation via qPCR of candidate miRNA within NHBE and DHBE derived sEVs: **F** miR411, **G** miR137, **H** miR195, **I** miR7. **J**–**M** Significantly differentially expressed miRNA showing links between miRNA and senescent genes and pathways based on Qiagen IPA data and literature. **J** miR411, **K** miR137, **L** miR195, **M** miR7. * and red points on volcano plot: p or p.adj < 0.05, Mann–Whitney test

Of the 551 miRNA present in either NHBEs or DHBE-derived sEVs, 377 of these were common to both sources (Fig. 5A). However, there are significantly differentially expressed miRNA profiles between NHBE and DHBE-derived sEVs with 19 significantly differentially expressed miRNAs found within DHBE-derived sEVs in comparison to NHBE-derived sEVs; 3 downregulated and 16 upregulated (Table 1) (n = 5–6; p.adj < 0.05) (Fig. 5B, C, Additional file 1: Fig. S4). This data indicates that the packaging of miRNA into sEVs differ depending on disease state, with those differential miRNA potentially responsible for the change in senescent phenotype that we see in our functional assays.

**Table 1:** Significantly differentially expressed miRNA (upregulated and downregulated) within DHBE-derived sEVs, compared to NHBE-derived sEVs

	Fold change
*Upregulated miRNA*	
hsa-miR-29a-3p	1.500711
hsa-miR-29c-3p	1.811905
hsa-miR-129-5p	2.563813
hsa-miR-411-5p	2.865036
hsa-miR-155-5p	1.441986
hsa-let-7c-5p	1.43471
hsa-miR-10b-5p	3.283013
hsa-miR-411-5p	2.580304
hsa-miR-195-5p	2.661103
hsa-miR-664a-5p	1.989499
hsa-miR-379-5p	1.735574
hsa-miR-137-3p	1.896443
hsa-miR-1262	2.833224
hsa-miR-190a-5p	3.376735
hsa-miR-132-3p	1.000998
hsa-miR-7-5p	1.641512
*Downregulated miRNA*	
hsa-miR-320a-3p	− 1.39872
hsa-miR-138-5p	− 2.29206
hsa-miR-7704	− 4.73151

N = 5

p.adj < 0.05

The 19 significantly differentially expressed miRNAs in DHBE-derived sEVs were taken through stringent ingenuity pathway analysis (IPA), to investigate miRNA-mRNA relationships and predict affected genes. Analysis of the most significantly affected pathways predicted by KEGG and GO analysis demonstrated that these miRNA were linked to multiple IPF and senescence pathways including regulation of the cell cycle, DNA damage response and p53 signalling pathway (Fig. 5D, E).

Of these differentially expressed miRNAs, 3 were confirmed by miR-qPCR to correlate with the differential expression profiles seen in the smRNA-seq dataset—miRNA-411, miRNA-195 and miRNA-7 (all n = 3; p < 0.05), with miRNA-137 showing a trend for increase (Fig. 5F–I). The analysis of this data is outlined in the methodology and absolute cT values shown in Additional file 1: Fig. S5. IPA, KEGG/GO pathway analysis and literature reviews predicted that each of these 4 miRNA affect cell proliferation, DNA damage and cell cycle pathways. Combined, they were predicted to modulate senescence related genes such as p53, p21 and sirt3 (Fig. 5J–M).

### miR411, miR137, miR195 and miR7 are upregulated in DHBEs, NHBEs and ALI cultures upon addition of DHBE-derived sEVs

**Fig. 6:**
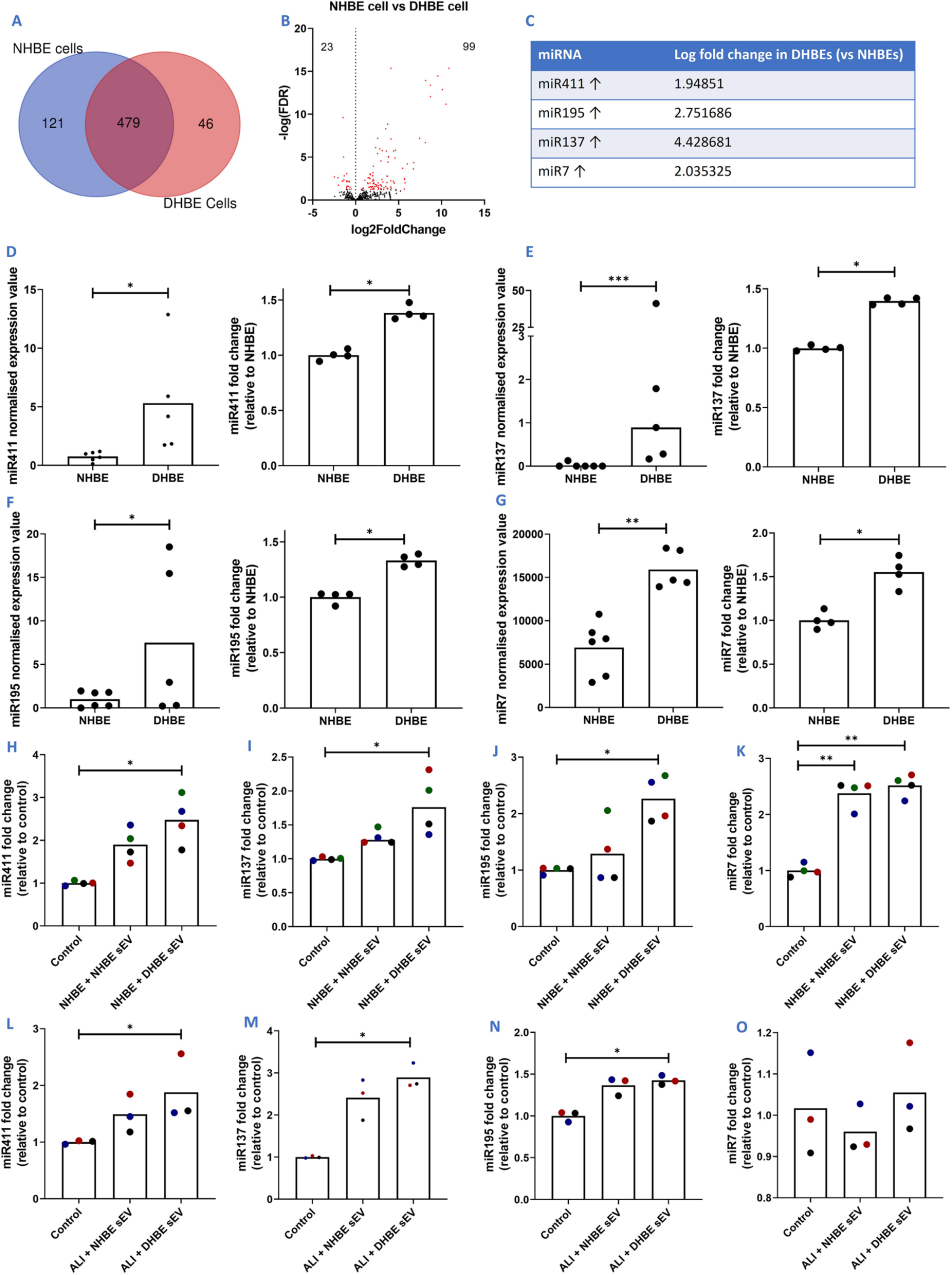
Candidate miRNAs are shown to be unregulated in DHBEs and responder cells/cultures co-cultured with diseased sEVs, indicating successful transfer via sEVs. **A** Overlap of miRNAs detected in NHBE and DHBE cells. **B** Volcano plot showing differential miRNA expression between NHBE and DHBE cells. **C** Fold change of candidate miRNA within DHBEs, compared to NHBEs. **D**–**G** smRNA-seq expression values and confirmation via qPCR of candidate miRNA: **E** miR411, **E** miR137, **F** miR195, **G** miR7. **H**–**K** Candidate miRNA expression within submerged responder cells co-cultured with NHBE and DHBE-derived sEVs: **H** miR411, **I** miR137, **J** miR195, **K** miR7. **L**–**O** Candidate miRNA expression within healthy ALI cultures co-cultured with NHBE and DHBE derived sEVs: **L** miR411, **M** miR137, **N** miR195, **O** miR7. Gene normalisation to RNU6B for δδCT and graphs show fold change relative to NHBEs or control of no added sEVs. N = 3-. Each colour represents a different donor and experiment. Significant differences between groups shown ***/red = p or p.adj < 0.001; **p < 0.01; *p < 0.05, Mann–Whitney test and Friedmans test

Of the 646 miRNA present in either NHBEs or DHBEs, 479 of these were common to both sources (Fig. 6A). A large number of miRNAs were differentially regulated between NHBEs and DHBEs, with 122 significantly differentially expressed miRNAs found in DHBEs, in comparison to NHBEs-23 downregulated and 99 upregulated (n = 5; p.adj < 0.05) (Fig. 6B). The four candidate miRNA, miR411, miR137, miR195 and miR7, which were upregulated in DHBE-derived sEVs were also upregulated in DHBE cells, compared to NHBE cells (Fig. 6C). This was confirmed via miR-qPCR (n = 4; p < 0.05) (Fig. 6D–G). Furthermore, all four of these miRNA were also upregulated in responder NHBE cells after co-culture with DHBE-derived sEVs (n = 4; p < 0.05) (Fig. 6H–K). Three of the four were also upregulated in healthy ALI cultures after co-culture with DHBE-derived sEVs (n = 3; p < 0.05) (Fig. 6L–O).

## Discussion

Within this study, we demonstrate the potential for sEVs from DHBEs to drive senescence in surrounding cells. Using primary epithelial cells from IPF patients and healthy controls, we have demonstrated that DHBEs release a higher number of sEVs compared to NHBEs and that these sEVs are actively taken up by recipient NHBEs within 24 h of co-culture. NHBEs co-cultured with DHBE-derived sEVs adopted a senescent phenotype. These DHBE-derived sEVs have a differentially expressed miRNA profile, in comparison to healthy controls which includes increased expression of miRNA-411, miRNA-137, miRNA-195, miRNA-7. The data obtained here suggests that sEVs play a key role in the propagation of senescence within IPF and that this transfer of sEVs from diseased epithelial cells to healthy cells may play an important role in disease pathogenesis.

We have normalised the amount of sEVs used in these experiments to decipher the effect of DHBE derived-sEV cargo compared to NHBE-derived sEVs [27, 29]. However, we have also noted an increased release of sEVs from DHBEs. In IPF patients, the combined effect of both the quantity and the differential cargo within DHBE-derived sEVs are factors that could potentially cause a much larger effect of DHBE-derived sEVs than what we have seen in vitro*.* Furthermore, we speculate that as the disease progresses and there are increases in diseased cells, there could be a widespread impact of sEVs which could further influence IPF disease state. We also know that sEVs must be intact to induce these effects, as once digested they did not induce the senescent phenotype that we had previously seen.

This is the first time that NHBEs and ALI cultures have been investigated as functional responder cells for the effect of DHBE-derived sEVs. As there is no single indicator of senescence, we have used multiple assays to detect senescent markers which covers SASP mediators, cell cycle regulators, DNA damage markers and SA-β-Gal. Co-culture experiments with IPF DHBE-derived sEVs show an increase of these senescent markers in NHBEs and healthy ALI cultures, alongside decreases in TEER measurements (indicating a change in the morphology of cells). There is an abundance of elevated senescence markers in IPF fibroblasts [36], something we also observed in isolated DHBEs (Additional file 1: Fig. S1). For the first time, we have elucidated that IPF DHBE-derived sEVs induces senescence in NHBEs, and may be involved in contributing to the senescent phenotype that evolves during IPF.

It is already known that IPF-derived sEVs from BALF and primary human lung fibroblasts are pro-fibrotic when co-cultured with lung fibroblasts [29] and pro-senescent, inducing mitochondrial damage and cell death when co-cultured with lung epithelial cells [27]. However, the initiating injury in IPF is believed to be epithelial and these are the first data that demonstrates DHBE-derived sEVs transfer senescence, possibly via their miRNA cargo. It is worth noting that where we see no effect (or at some points a non-significant slightly detrimental effect) with NHBE-derived sEVs, which differs from previous research, the in vitro assay for these experiments differs in that we use unstimulated NHBEs, not TGFβ stimulated cells [30]. Despite these finding, the extent to which each cell type contributes to the pathogenesis of IPF is yet to be elucidated.

We demonstrated that there is differential smRNA expression both between sEVs and their parental cells and between NHBE and DHBE-derived sEVs. In the first instance, differences between sEVs and their parental cells indicate that there may be an active and selective packaging mechanism for these sEVs. However, it should be noted that of the nineteen miRNAs that were significantly differentially expressed between DHBE-derived sEVs and NHBE-derived sEVs, the four (miRNA-411, miRNA-137, miRNA-195, miRNA-7) that were confirmed by qPCR to be upregulated in DHBE-derived sEVs, were also increased in DHBEs compared to NHBEs. The possibility cannot be dismissed that at least in the case of these miRNAs, levels may also be dependent on enrichment of these miRNA in parental cells. Differential expression between NHBE and DHBE-derived sEVs also confirms that cargo within sEVs differs depending on diseased state and these differences could be a factor in the senescent changes we see in our functional data. Furthermore, it would be interesting to determine whether these miRNA could also be detected in the circulation of IPF patients, as it has previously been shown that miRNAs that are differentially expressed in the sputum and serum of IPF patients correlated with lung function and survival analysis and have been investigated as potential biomarkers [25, 26].

Employing tagged RNA we demonstrated sEVs were taken up by recipient cells. Of our candidate miRNA, four were upregulated in submerged NHBEs and three in ALI cultures upon incubation with DHBE-derived sEVs and caused a change in recipient cell phenotype. Together this is indicative of successful transfer of miRNAs which induce an effect. These data lead us to hypothesise that these increases in miRNA are due to both the transfer of miRNA cargo within the sEVs and the consequence of sEV induced phenotypic changes in the recipient cells triggering higher levels of miRNA expression.

The differences between NHBE and DHBE sEV release, cargo and effect on responder cells suggest that bronchial epithelial cells play an important role in IPF. Bronchial epithelial cells have been reported to contribute to IPF relevant pathways, including TGF-β [38] and the Wnt signalling pathway [39], which we also see as predictive pathways in our KEGG/GO analysis. Others have also demonstrated ex vivo that alveolar epithelial cells from fibrotic tissue have a senescent phenotype and secrete proinflammatory and profibrotic molecules [37]. Similarly, this study has confirmed that DHBEs have a more senescent phenotype, compared to NHBEs in vitro (Additional file 1: Fig. S1), and that there are differences in DHBE secretome, including secreted sEVs, which are suggested to affect markers and pathways associated with the senescent pathway. Future work could also focus on extending these experiments to investigate small airway epithelial cells (SAECs), to determine whether this is a consistent effect between airway and alveolar epithelial cells. However, accessing and isolating these samples from diseased tissue are challenging in comparison to bronchial brushings, with experimental isolation yielding a small amount of cells. Importantly, it has been noted that healthy SAECs and NHBEs reacted similarly to fibroblast-derived sEVs [27] and that healthy SAECs-derived sEVs and NHBE-derived sEVs both reduce senescence in NHBEs stimulated with TGF-β [30], indicating similar functionality between the two sources.

There are a number of limitations to our work. sEVs contain other types of smRNA as identified through RNA-sequencing and it is not yet known whether these, alongside other proteins which are present within sEVs, may contribute towards the pathological effect that we see here. A large amount of sEVs are required from isolation to see an effect within our single treatment experiments. However, we hypothesise that within patients, continuous release of smaller amounts of sEVs could be sufficient to induce an effect. Furthermore, the miRNA that we identified for evaluation are predicted to impact genes associated with multiple pathways beyond senescence and these sEVs could play a role in IPF through pathways such as Wnt signalling and fibrosis. Future work will therefore focus on using miRNA mimics to confirm the effect of these miRNAs on senescence and further determine specific mechanisms of action.

To conclude, we have identified a novel method of senescence propagation to NHBEs mediated by DHBE-derived sEVs. These sEVs induce senescence within their microenvironment and may cause a feed-forward cycle that circulates a wave of senescence across the epithelium. Differential miRNA cargo within these sEVs could be the cause of this induced senescence in both submerged and ALI cultures of healthy epithelial cells and miRNAs identified within this study (miR411, miR137, miR195 and miR7) may highlight pathways for novel therapeutics to target within IPF.

## Supplementary Information

**Additional file 1: Figure S1.** DHBEs have a greater expression of senescent markers, compared to NHBEs (A-D) Increased SASP release including IL6, IL8, PAI1 and TNF-a. (E-J) Increased senescence related gene expression including p16, p21, p27, p15, Sirt1 and Sirt6. (K) Increased γH2AX positive diseased cells (L) Decreased cell count/proliferation from DHBEs. N=3-5. Each colour represents a different donor and experiment. Significant differences between groups shown ***=p<0.001**=p<0.01; *=p<0.05. Mann-Whitney test. **Figure S2.** Incubation with Triton X as a detergent to breakdown vesicles and release contents shows loss of effect seen with intact vesicles. (A) γH2AX (B) IL6 (C) IL8 (D) p16 gene expression (E) p21 gene expression. Control used is with no added sEVs. N=3-5. ***=p<0.001; **=p<0.01; *=p<0.05. Wilcoxon test. **Figure S3.** TEER data (Figure 3G) in ohms (cm^2^). Control used is with no added sEVs. N=3-5. ***=p<0.001; **=p<0.01; *=p<0.05. Friedmans test. **Figure S4.** Expression values of 19 significantly differentially expressed (p<0.05) miRNA from smRNA-seq (Figure 5B). N=5-6. **Figure S5.** CT values of candidate miRNAs (Figure 5F-I) from confirmation qPCRs. N=3. **Table S1.** Cell source, patient characterisation and demographics. **Table S2.** Antibodies used for EV protein blotting. **Table S3.** Primers used for qPCR confirmation of candidate miRNAs.

## Data Availability

Data underlying the findings described in this manuscript may be obtained in accordance with AstraZeneca’s data sharing policy described at https://astrazenecagrouptrials.pharmacm.com/ST/Submission/Disclosure. Use the “Enquiries about Vivli Member Studies” (https://vivli.org/members/enquiries-about-studies-not-listed-on-the-vivli-platform/) form and include the publication title and data Accession number GSF1239080 in your request.

## Abbreviations

sEVs: Small extracellular vesicles
IPF: Idiopathic pulmonary fibrosis
NHBEs: Normal human bronchial epithelial cells
DHBEs: IPF-diseased human bronchial epithelial cells
miRNA: MicroRNA
TEER: Transepithelial electrical resistance
ALI: Air liquid interface
SASP: Senescence-associated secretory phenotype
BALF: Bronchoalveolar lavage fluid
PBS: Phosphate-buffered saline
SA-β-Gal: Senescence-associated beta-galactosidase
NTA: Nanoparticle tracking analysis
smRNA: Small ribonucleic acid
IPA: Ingenuity pathway analysis
TEM: Transmission electron microscopy
ECM: Extracellular matrix
TGFß: Transforming growth factor-beta
UC: Ultracentrifugation
PCA: Principle component analysis
P.adj: Adjusted p value
