# Supplementary material for: Epithelial senescence in idiopathic pulmonary fibrosis is propagated by small extracellular vesicles
Source: Respir Res. 2023 Feb 14;24:51. doi: 10.1186/s12931-023-02333-5 (PMC9930250; doi:10.1186/s12931-023-02333-5)

Figure S1: DHBEs have a greater expression of senescent markers, compared to NHBEs (A-D) Increased SASP release including IL6, IL8, PAI1 and TNF-a. (E-J) Increased senescence related gene expression including p16, p21, p27, p15, Sirt1 and Sirt6. (K) Increased γH2AX positive diseased cells (L) Decreased cell count/proliferation with diseased epithelial cells. N=3-5. Each colour represents a different donor and experiment. Significant differences between groups shown ***=p<0.001**=p<0.01; *=p<0.05. Mann-Whitney test.

Figure S2: Incubation with Triton X as a detergent to breakdown vesicles and release contents shows loss of effect seen with intact vesicles. (A) γH2AX (B) IL6 (C) IL8 (D) p16 gene expression (E) p21 gene expression. Control used is with no added sEVs. N=3-5. ***=p<0.001; **=p<0.01; *=p<0.05. Wilcoxon test.

Figure S3: TEER data (Figure 3G) in ohms (cm^2^). Control used is with no added sEVs. N=3-5. ***=p<0.001; **=p<0.01; *=p<0.05. Friedmans test.

Figure S4: Expression values of 19 significantly differentially expressed (p<0.05) miRNA from smRNA-seq (Figure 5B). N=5-6.

Figure S5: CT values of candidate miRNAs (Figure 5F-I) from confirmation qPCRs. N=3.

Table S1: Cell source, patient characterisation and demographics

|  | NHBEs (n=7) | IPF DHBEs (n=8) |
| --- | --- | --- |
| Age, years mean (± SD) | 57.1 (± 8.9) | 65.6 (± 7.2) |
| Males. % of group | 71.4% | 75.0% |
| Ex- or current-smoker (%) | 42.8% | 50% |
| FVC, % predicted (± SD) | N/A | 81.5% (± 20.15) |

Data are presented as percentage value or mean ± standard deviation (SD) as appropriate.

FVC: forced vital capacity.

Table S2: Antibodies used for EV protein blotting

| Name | Concentration use | Source |
| --- | --- | --- |
| Alix (3A9) Mouse mAb #2171 | 1:1000 | Mouse IgG |
| Annexin V Antibody #8555 | 1:1000 | Rabbit IgG |
| CD54/ICAM-1 Antibody #4915 | 1:1000 | Rabbit IgG |
| CD9 (D8O1A) Rabbit mAb #13174 | 1:1000 | Rabbit IgG |
| GM130 (D6B1) XP^®^ Rabbit mAb #12480 | 1:1000 | Rabbit IgG |
| EpCAM (D1B3) Rabbit mAb #2626 | 1:1000 | Rabbit IgG |
| HSP70 (D69) Antibody #4876 | 1:1000 | Rabbit IgG |
| Flotillin-1 (D2V7J) XP^®^ Rabbit mAb #18634 | 1:1000 | Rabbit IgG |
| Anti-rabbit IgG, HRP-linked Antibody #7074 | 1:1000 |  |
| Anti-mouse IgG, HRP-linked Antibody #7076 | 1:1000 |  |

Table S3: Primers used for qPCR confirmation of candidate miRNAs

| Name | Assay ID | Catalogue number |
| --- | --- | --- |
| hsa-miR-411-5p | [478086_mir](https://www.thermofisher.com/order/genome-database/details/microrna/478086_mir?CID=&ICID=&subtype=) | [A25576](https://www.thermofisher.com/order/catalog/product/A25576) |
| hsa-miR-7-5p | [483061_mir](https://www.thermofisher.com/order/genome-database/details/microrna/483061_mir?CID=&ICID=&subtype=) | [A25576](https://www.thermofisher.com/order/catalog/product/A25576) |
| hsa-miR-195-5p | [477957_mir](https://www.thermofisher.com/order/genome-database/details/microrna/477957_mir?CID=&ICID=&subtype=) | [A25576](https://www.thermofisher.com/order/catalog/product/A25576) |
| hsa-miR-138-5p | [477905_mir](https://www.thermofisher.com/order/genome-database/details/microrna/477905_mir?CID=&ICID=&subtype=) | [A25576](https://www.thermofisher.com/order/catalog/product/A25576) |
| hsa-miR-132-3p | [477900_mir](https://www.thermofisher.com/order/genome-database/details/microrna/477900_mir?CID=&ICID=&subtype=) | [A25576](https://www.thermofisher.com/order/catalog/product/A25576) |
| hsa-miR-320a-3p | [478594_mir](https://www.thermofisher.com/order/genome-database/details/microrna/478594_mir?CID=&ICID=&subtype=) | [A25576](https://www.thermofisher.com/order/catalog/product/A25576) |
| hsa-miR-10b-5p | [478494_mir](https://www.thermofisher.com/order/genome-database/details/microrna/478494_mir?CID=&ICID=&subtype=) | [A25576](https://www.thermofisher.com/order/catalog/product/A25576) |
| hsa-miR-129-5p | [477896_mir](https://www.thermofisher.com/order/genome-database/details/microrna/477896_mir?CID=&ICID=&subtype=) | [A25576](https://www.thermofisher.com/order/catalog/product/A25576) |
| hsa-miR-137-3p | 477904_mir | [A25576](https://www.thermofisher.com/order/catalog/product/A25576) |
| hsa-miR-190a-5p | [478358_mir](https://www.thermofisher.com/order/genome-database/details/microrna/478358_mir?CID=&ICID=&subtype=) | [A25576](https://www.thermofisher.com/order/catalog/product/A25576) |
| hsa-miR-379-5p | [478077_mir](https://www.thermofisher.com/order/genome-database/details/microrna/478077_mir?CID=&ICID=&subtype=) | [A25576](https://www.thermofisher.com/order/catalog/product/A25576) |
| hsa-miR-29c-3p/hsa-miR-29a-3p | [479229_mir](https://www.thermofisher.com/order/genome-database/details/microrna/479229_mir?CID=&ICID=&subtype=)/[478587_mir](https://www.thermofisher.com/order/genome-database/details/microrna/478587_mir?CID=&ICID=&subtype=) | [A25576](https://www.thermofisher.com/order/catalog/product/A25576)/[A25576](https://www.thermofisher.com/order/catalog/product/A25576) |


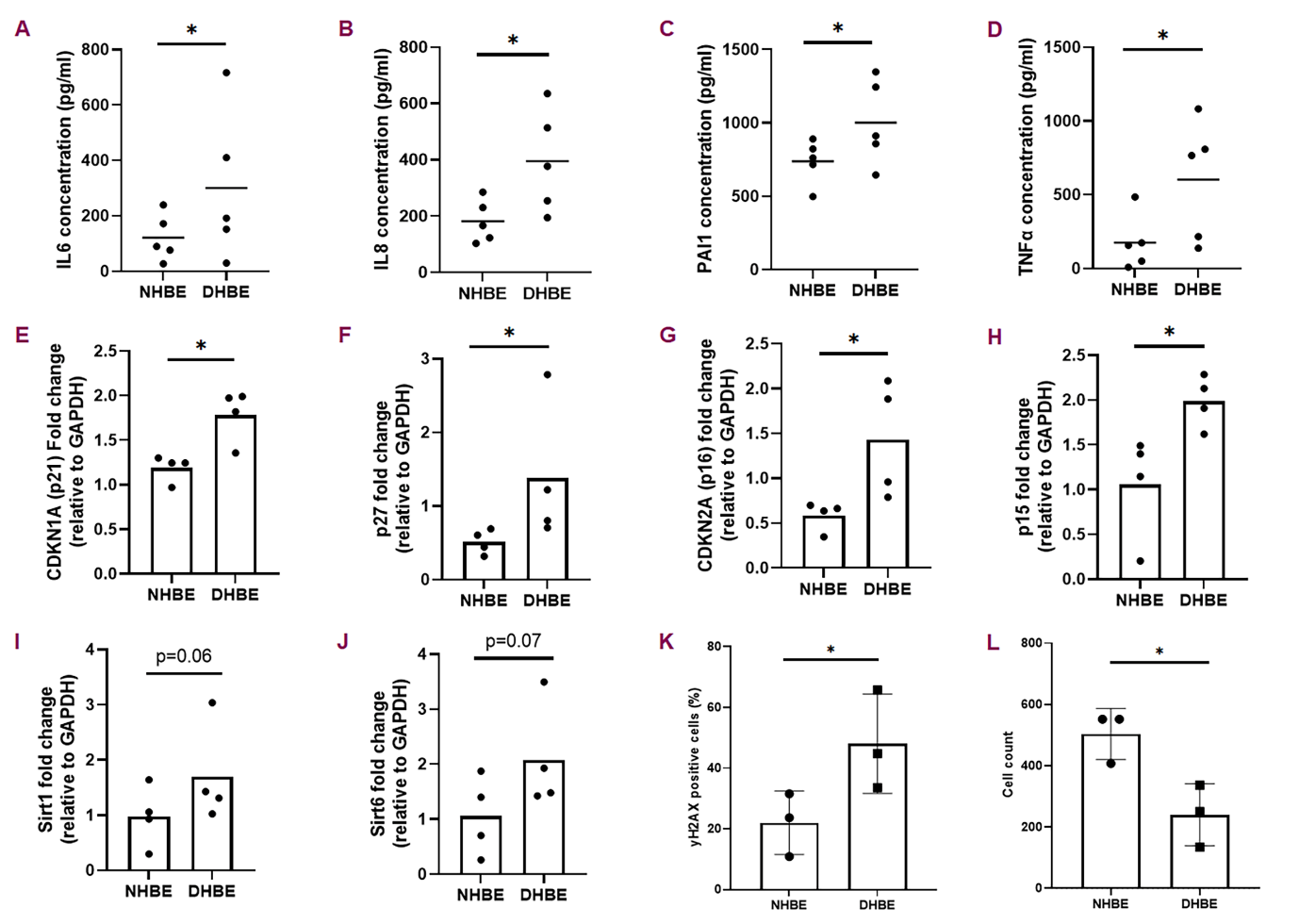


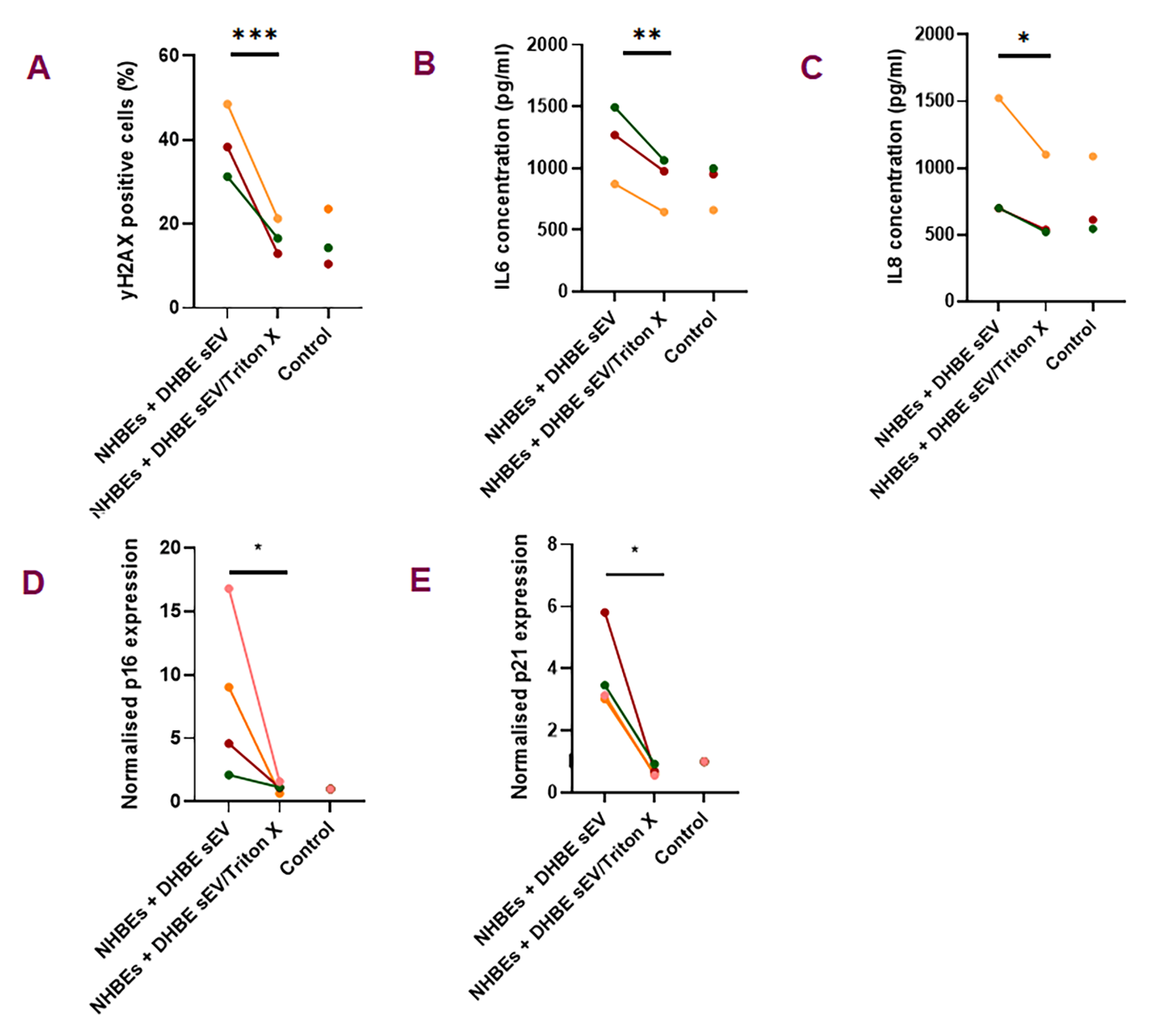


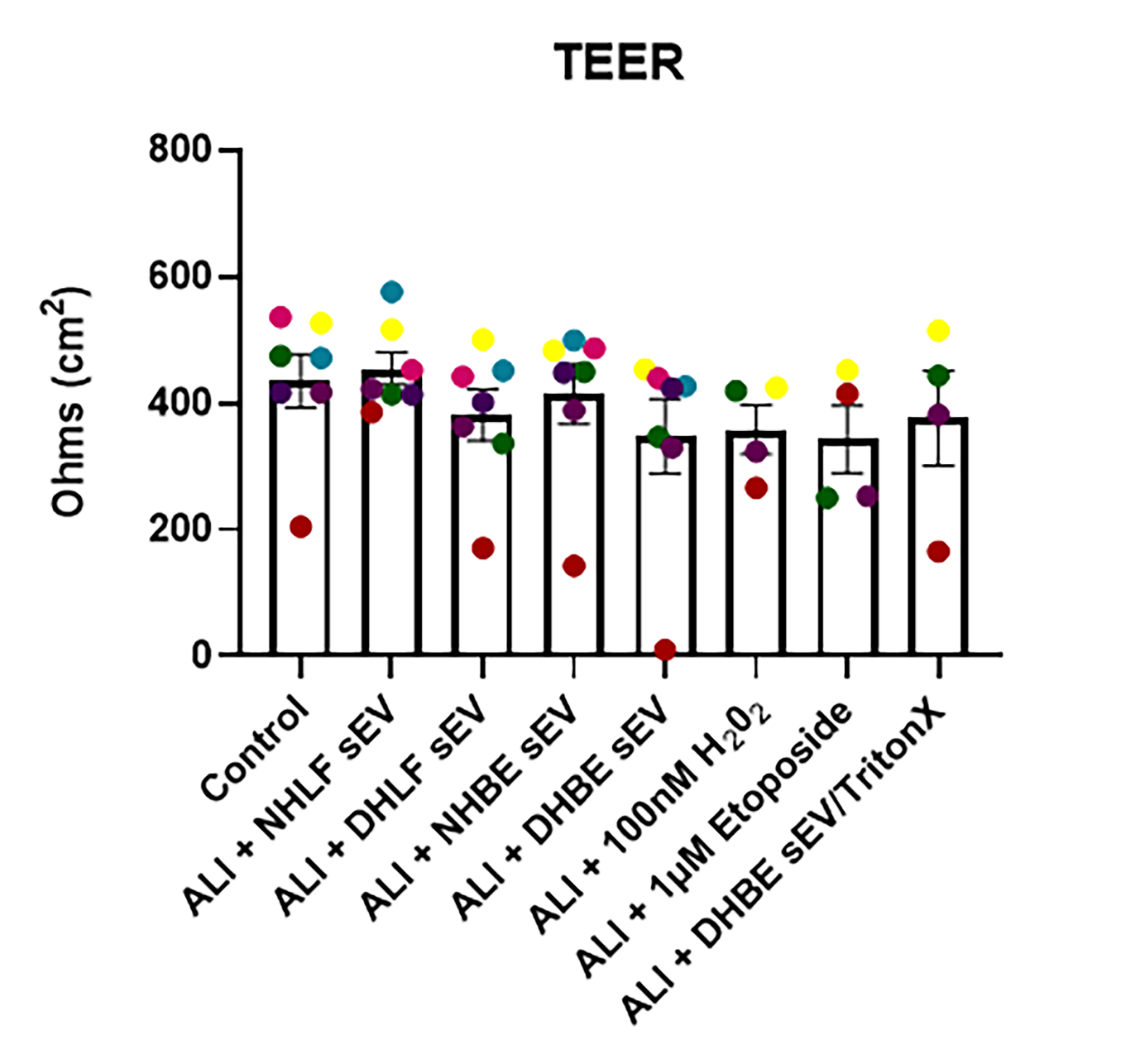


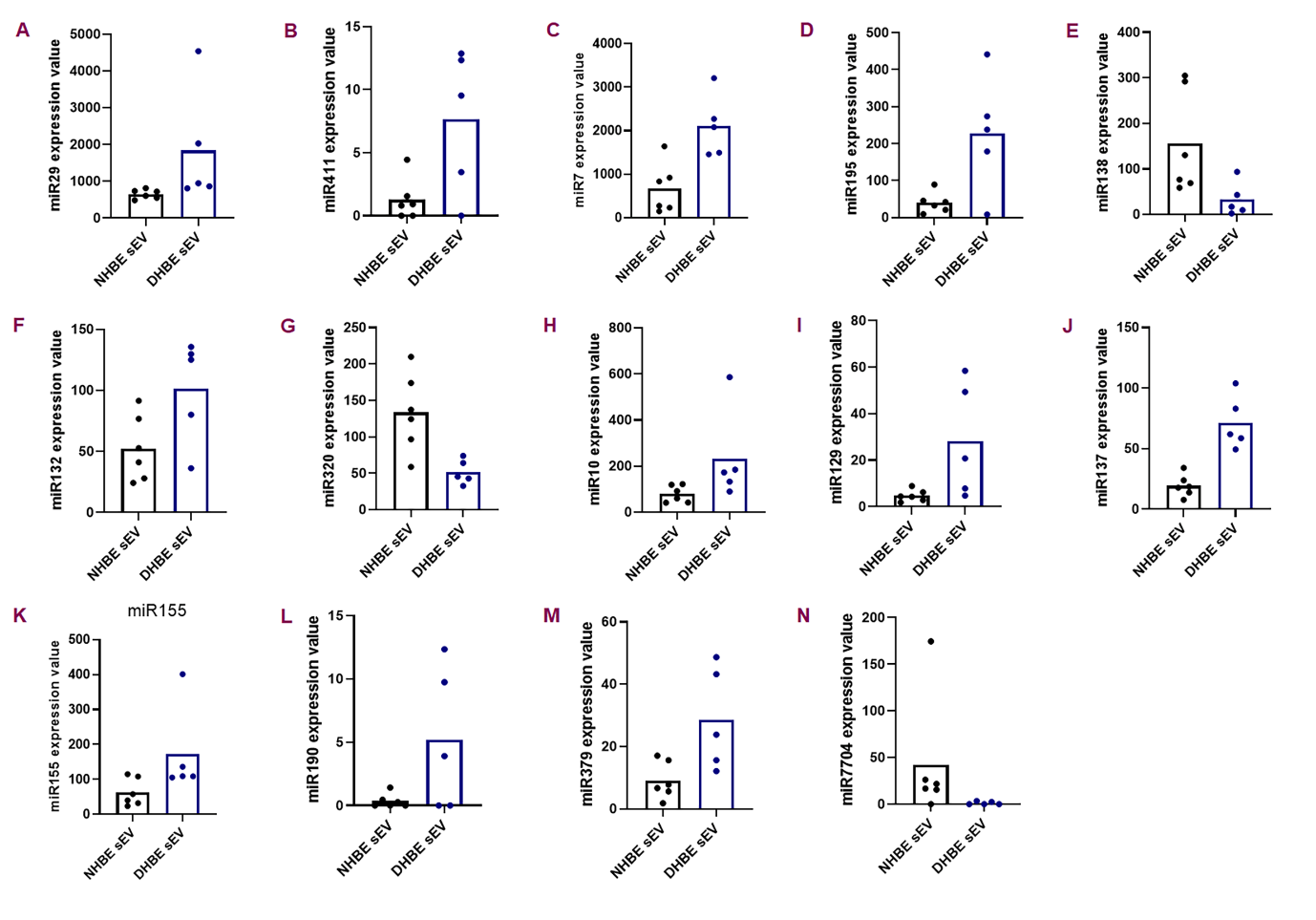


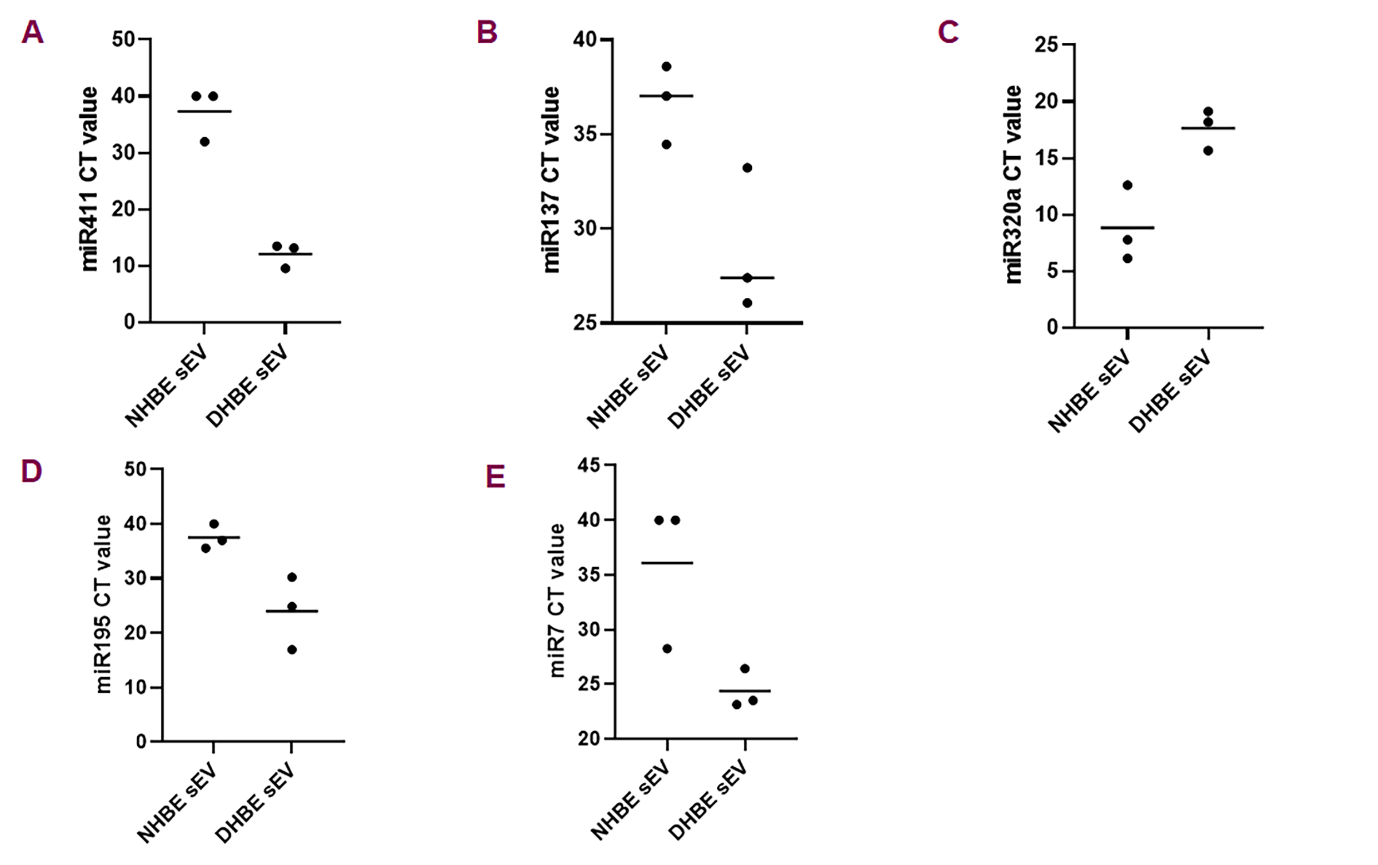

Supplement: Supplementary file 1 — Additional file 1: Figure S1. DHBEs have a greater expression of senescent markers, compared to NHBEs (A-D) Increased SASP release including IL6, IL8, PAI1 and TNF-a. (E-J) Increased senescence related gene expression including p16, p21, p27, p15, Sirt1 and Sirt6. (K) Increased γH2AX positive diseased cells (L) Decreased cell count/proliferation from DHBEs. N=3-5. Each colour represents a different donor and experiment. Significant differences between groups shown ***=p<0.001**=p<0.01; *=p<0.05. Mann-Whitney test. Figure S2. Incubation with Triton X as a detergent to breakdown vesicles and release contents shows loss of effect seen with intact vesicles. (A) γH2AX (B) IL6 (C) IL8 (D) p16 gene expression (E) p21 gene expression. Control used is with no added sEVs. N=3-5. ***=p<0.001; **=p<0.01; *=p<0.05. Wilcoxon test. Figure S3. TEER data (Figure 3G) in ohms (cm2). Control used is with no added sEVs. N=3-5. ***=p<0.001; **=p<0.01; *=p<0.05. Friedmans test. Figure S4. Expression values of 19 significantly differentially expressed (p<0.05) miRNA from smRNA-seq (Figure 5B). N=5-6. Figure S5. CT values of candidate miRNAs (Figure 5F-I) from confirmation qPCRs. N=3. Table S1. Cell source, patient characterisation and demographics. Table S2. Antibodies used for EV protein blotting. Table S3. Primers used for qPCR confirmation of candidate miRNAs. [file 12931_2023_2333_MOESM1_ESM.docx]
